# A mixed analysis comparing nine minimally invasive surgeries for unresectable hepatocellular carcinoma patients

**DOI:** 10.18632/oncotarget.12348

**Published:** 2016-09-29

**Authors:** Ran Tao, Xiaodan Li, Ruizhi Ran, Zhihua Xiao, Hongyue Zhang, Hongyan Kong, Qiqin Song, Yu Huang, Likui Wang, Jiaquan Huang

**Affiliations:** ^1^ Department and Institute of Infectious Disease, Tongji Hospital, Tongji Medical College, Huazhong University of Science and Technology, Wuhan, Hubei, China; ^2^ Department of Infectious Diseases,The Central Hospital of Enshi Autonomous Prefecture, Enshi Clinical College of Wuhan University, Wuhan, Hubei, China; ^3^ Department of Interal Medicine-Oncology, The Central Hospital of Enshi Autonomous Prefecture, Enshi Clinical College of Wuhan University, Wuhan, Hubei, China; ^4^ Department of Medical Oncology, Hubei Cancer Hospital, Wuhan, Hubei, China; ^5^ Savaid Medical School, University of Chinese Academy of Sciences Institute of Microbiology, Chinese Academy of Sciences, Beijing, China

**Keywords:** unresectable hepatocellular carcinoma, transcatheter arterial chemoembolization, network meta-analysis

## Abstract

Hepatocellular carcinoma (HCC) is usually managed by the transcatheter arterial chemoembolization (TACE). However, this technique has been challenged since severe complications have been observed in clinical practices. As a result, clinicians have started to seek other minimally invasive surgeries with equivalent efficacy. The corresponding surgeries were assessed by the five outcomes: complete response (CR), partial response (PR), stable disease (SD), progression disease (PD) and objective response rate (ORR). Direct meta-analysis and network meta-analysis were performed and the results were represented by odds ratios (OR), 95% confidence and credential intervals. Furthermore, the value of surface under the cumulative ranking curve (SUCRA)was calculated to provide corresponding rankings.Seventeen studies were incorporated into the network meta-analysis which indicated that TACE + external-beam radiation therapy (EBRT) and drug-eluting beads (DEB) were better than TACE at controllingPD. TACE + EBRT demonstrated their advantages compared to TARE-90Y.However, network meta-analysis comparison showed no significant difference between the corresponding eight treatments with respect to CR, PR, SD and ORR. Moreover, the SUCRA suggested that TACE+EBRT were better than other treatments at treating unresectableHCC.Based on the present results of this network meta-analysis, TACE + EBRT was more effective than the other seven minimally invasive surgeries and therefore it is considered as the optimal treatment for HCC.

## INTRODUCTION

Hepatocellular carcinoma (HCC)is one of the most lethal cancers all around the world. New medications and treatments are being developed to tackle the high morbidity and mortality of HCC [1].Currently, resection has been the first choice for managing HCC at early stages [2]. However, early symptoms of HCC are difficult to be identified due to the specificity of HCC. As a result, HCC is usually diagnosed at advanced stages in which conventional treatments such as local ablation therapy, surgical resection and liver transplantation are not feasible [3].

A previous study suggested that transcatheter arterial chemoembolization (TACE) is the first choice to treat the intermediate-stage HCC [4]. The proportion of HCC patients who achieved partial remission after TACE is approximately 62% and both tumor progression and vascular invasion were delayed significantly [5]. As suggested by various randomized controlled trials, TACE improved the average survival time by approximately two years compared to symptomatic treatments and systemic chemotherapies [6].Common techniques including sorafenib (SOR), percutaneous ethanol injection (PEI), high intensity focused ultrasound (HIFU), drug-eluting beads (DEB) and external-beam radiation therapy (EBRT) can be combined with other therapies. Furthermore, studies have provided evidence that introducing HIFU into TACE improved the overall survival rate compared to TACE alone [7] and the cumulative survival rate of HCC patients treated by PEI in conjunction with TACE was 12.7% higher than those treated with TACEalone [8].Some studies also suggested that introducing DEB into TACE enhanced the effectiveness of TACE and such an enhanced effectiveness is reflected by an improved survival rate as well as theinhibition of tumor progression [9].Although there was no significant difference in the total survival rate between the ethanol ablation (TEA) and TACE, it is acknowledged that TEA has more durable effects on HCC patients than TACE [10]. Recently, the yttrium-90 radio embolization (TARE-90Y)has started to replace TACEwith respect to local HCC [4].

The elegant approach of network meta-analysis enabled us to compare the efficacy of different minimally invasive surgeries by synthesizing both direct and indirect evidence obtained from randomized trials. Apart from that, the network meta-analysis provided us with a comprehensive ranking with respect to different endpoints and such a ranking may be used to distinguishing one treatment from another [11].

## RESULTS

### Baseline characteristics of the included studies

**Figure 1:**
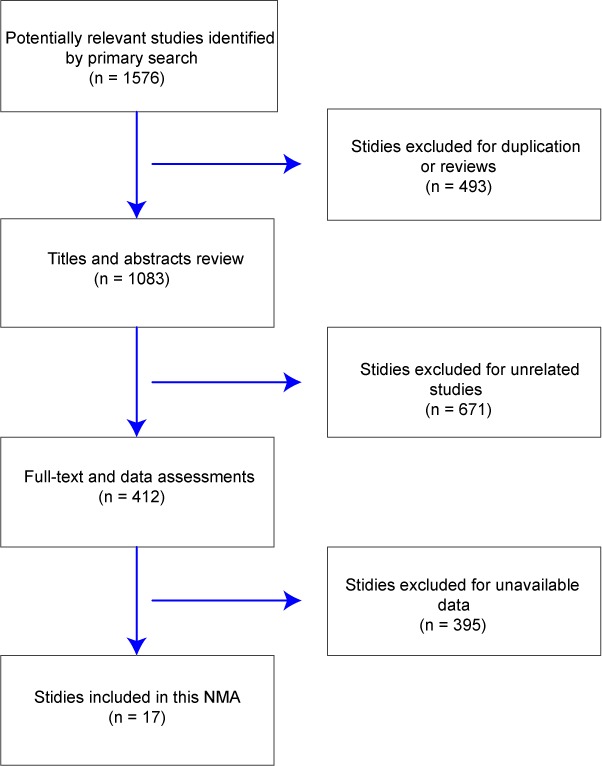
Literature selection flow chart

Initially, 1,576potentially relevant studies were identifiedfrom databases by using the predefined protocol. A total of 493studieswere excluded for duplicate publicationsor reviews, leaving 1,083 studies which passed the title and abstract review. We further excluded 671 studies since they either were not relevant to our research areas or were not qualified for the exclusion criteria. Another 395 studies were excluded as the full-text or the corresponding data were not available. Finally,17 studies were incorporated into the network meta-analysis (Figure 1) [8–10, 12–25]. Among a total of 2,669 patients with untreated unresectable HCC,1,560 patients from 16 studies were treated with TACE, 256 patients from 3 studies were treated with TACE+SOR, 44 patients from 1 study were treated with TACE+HIFU, 27 patients from 1 study were treated with TACE+PEI, 236 patients from 5 studies were treated with DEB-TACE, 230 patients from4 studies were treated with TARE-90Y, 191 patients from 1 study were treated with SOR, 54 patients from 1 study were treated with TACE+EBRT, and 45 patients from 1 study were treated with TEA. The 17 correspondingstudies were published between 1995 and 2014, all of which are two-arm trials. The baseline characteristics of the incorporated studies were summarized in Table 1.

**Table 1:** Main characteristics of included studies

Study	Region	Year	Participant		Treatment		Size		Endpoints
			Age (SD)	Male (%)	1	2	1	2	
01 Yu (2014)	Hong Kong	2014	65±20	80	TEA	TACE	45	45	➀➁➄
02 Wei Bai (2013)	China	2013	52±12	89	TACE+SOR	TACE	82	164	➁➂➃➄
03 Moreno-Luna (2013)	United States	2013	65±15	79	TARE-90Y	TACE	61	55	➀➁➂➃➄
04 Song (2012)	Korea	2012	60±11	70	DEB-TACE	TACE	60	69	➀➁➂➃➄
05 Song (2011)	Korea	2011	64±9	78	DEB-TACE	TACE	20	20	➀➁➂➄
06 Wiggerman (2011)	Germany	2011	68±9	84	DEB-TACE	TACE	22	22	➁➂➃➄
07 Malagari (2011)	Greece	2011	70±8	77	DEB-TACE	TACE	41	43	➁➂➃➄
08 Li (2010)	China	2010	49±8	80	TACE+HIFU	TACE	44	45	➀➁➂➃➄
09 Tan (2010)	China	2010	45±7	100	TACE+SOR	TACE	10	10	➁➂➃➄
10 Kooby (2010)	United States	2010	60±11	83	TARE-90Y	TACE	27	44	➁➂➃➄
11 Carr (2010)	United States	2010	-	-	TARE-90Y	TACE	99	691	➀➁➂➃➄
12 Lammer (2010)	Austria	2010	67±9	82	DEB-TACE	TACE	93	108	➀➁➂➃➄
13 Lewandowski	United States	2009	67±7	86	TARE-90Y	TACE	43	43	➁➂➃➄
14 Becker (2005)	Germany	2005	64±8	79	TACE+PEI	TACE	27	25	➁➂➃➄
15 Zeng (2004)	China	2004	51±12	91	TACE+EBRT	TACE	54	149	➀➁➂➃➄
16 Bartolozzi (1995)	Italy	1995	65±6	77	TACE+PEI	TACE	26	27	➀ ➃➄

* CR: complete response; PR: partial response; SD: stable disease; PD: progression disease; ORR: objective response rate; DEB-TACE: drug-eluting beads-transcatheter arterial chemoembolization; SOR: sorafenib; TACE: transcatheter arterial chemoembolization; EBRT: external-beam radiation therapy; HIFU: high intensity focused ultrasound; PEI: percutaneous ethanol injection; TARE-90Y: yttrium-90 radioembolization; TEA: transarterial ethanol ablation.

### Direct meta-analysis

**Figure 2:**
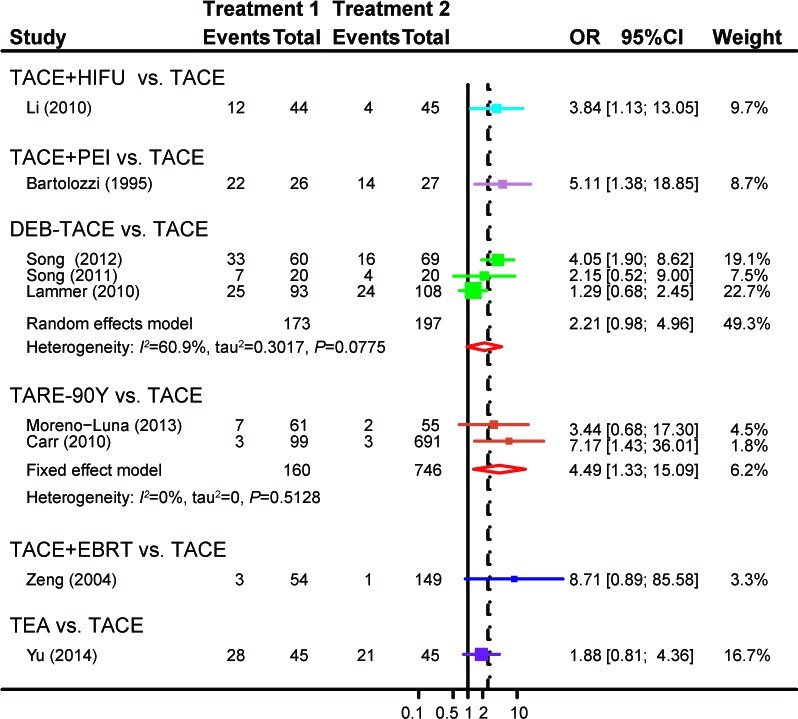
Forest plot of CRbyusingthe pair-wise meta-analysis

**Figure 3:**
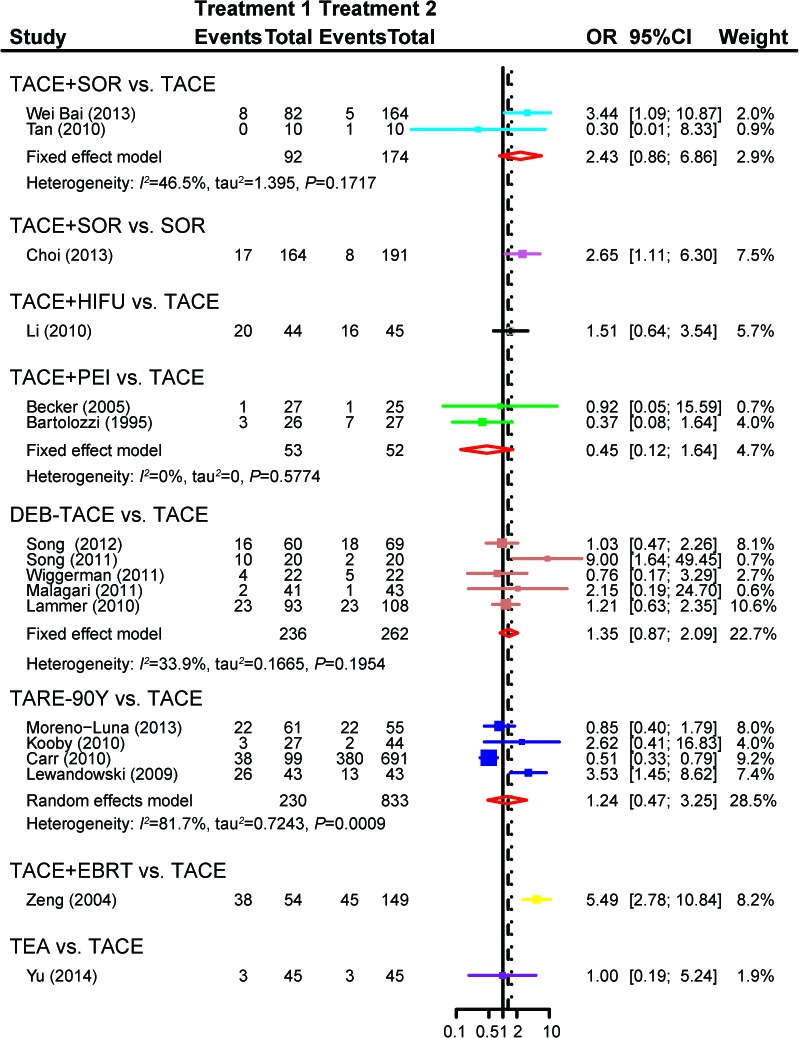
Forest plot of PRby usingthe pair-wise meta-analysis

**Figure 4:**
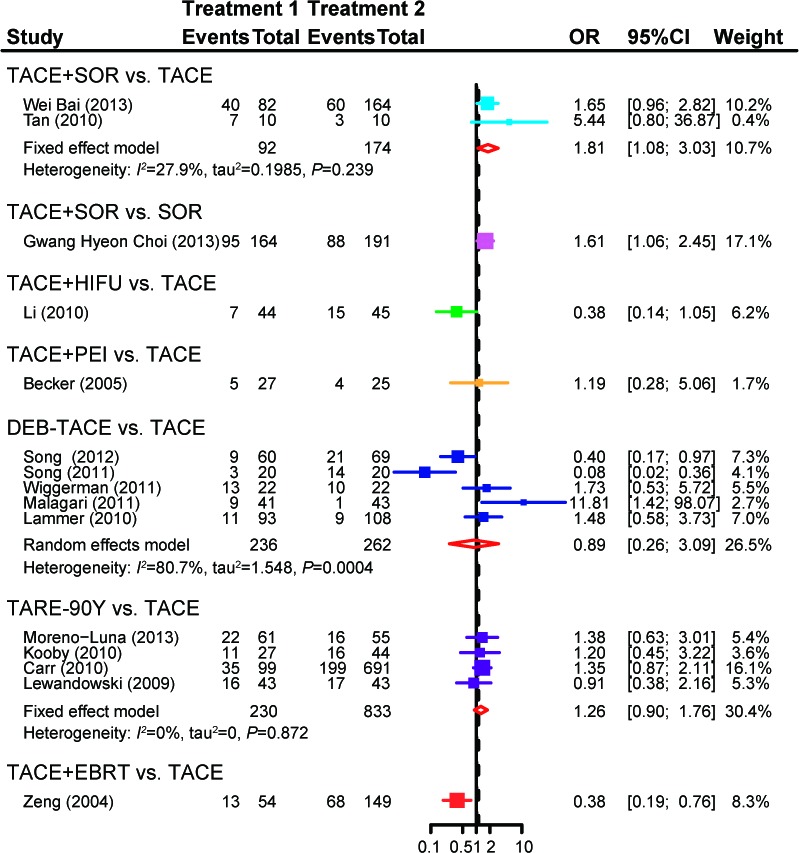
Forest plot of SDby using the pair-wise meta-analysis

The effectiveness of the eight treatments was compared in direct meta-analysis. As compared to TACE, TACE+HIFU(OR = 3.84, 95%CI = 1.13-13.05), TACE+PEI(OR = 5.11, 95%CI = 1.38-18.85) and TARE-90Y(OR = 4.49, 95%CI = 1.33-15.09)were more likely to achieve aCR among patients with unresectableHCC. By contrast, DEB-TACE (OR = 2.21, 95%CI = 0.98-4.96), TACE+EBRT (OR = 8.71, 95%CI = 0 .89-85.58) and TEA (OR = 1.88, 95%CI = 0.81-4.36) appeared to have similar effects with TACE on patients (Figure 2). Moreover, TACE+EBRT (OR = 5.49, 95%CI = 2.78-10.84) were more likely to induce PR compared to TACE (Figure 3). Besides that, TACE+SOR(OR = 1.81, 95%CI = 1.08-3.03)appeared to have stronger effects than SOR with respect to stabilizing the condition of patients (Figure 4).

**Figure 5:**
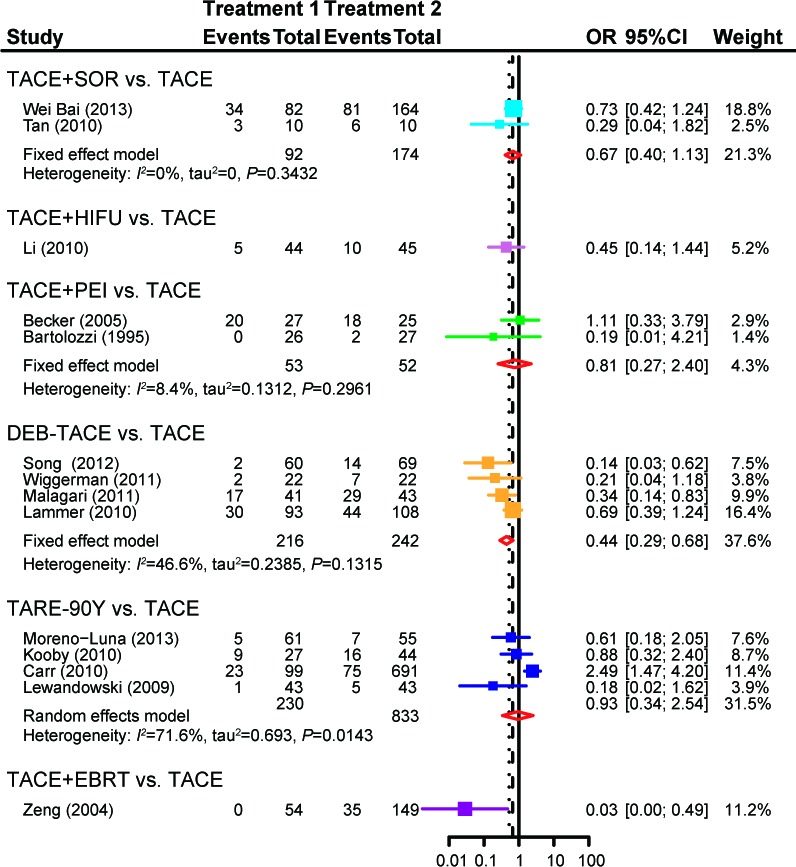
Forest plot of PDby using the pair-wise meta-analysis

**Figure 6:**
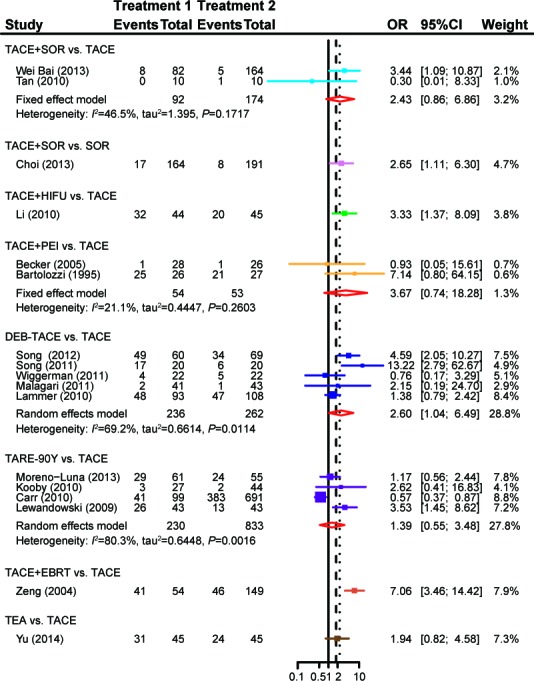
Forest plot of ORRby using thepair-wise meta-analysis

When compared with TACE, ACE+SOR (OR = 0.67, 95%CI = 0.40-1.13), TACE+HIFU (OR = 0.45, 95%CI = 0.14-1.44), TACE+PEI (OR = 0.81, 95%CI = 0.27-2.40) and TARE-90Y (OR = 0.93, 95%CI = 0.34-2.54) showed no significant difference in the probability of PD, while patients treated with DEB-TACE (OR = 0.44, 95%CI = 0.29-0.68) or TACE+EBRT (OR = 0.03, 95%CI = 0.01-0.49) were less likely to experiencePD (Figure 5). Treatments including TACE+HIFU (OR = 3.33, 95%CI = 1.37-8.09), DEB-TACE (OR = 2.60, 95%CI = 1.04-6.49)and TACE+EBRT (OR = 7.06, 95%CI = 3.46-14.42) exhibited enhanced effectiveness compared to TACE with respect to ORR(Figure 6).

### Network meta-analysis

As suggested by the network meta-analysis, both TACE+EBRT and DEB-TACE(OR = 0.04, 95%Crl = 0.01-0.52; OR = 0.33, 95%Crl = 0.09-0.94)are more effective than TACE with respect to the inhibition of PD. Apart from that, TACE+EBRT exhibited further superiority over TARE-90Y(OR = 0.04, 95%Crl = 0.00-0.82; Table 5). However, network meta-analysis comparison indicated no significant difference in CR, PR, SD and ORR amongtreatments (Tables 2, 3, 4, 6).

**Table 2:** CR of seven minimally invasive surgeries in unresectable HCC according to the network meta-analysis using odds ratios (ORs) and corresponding 95% credible intervals (CrI)

**DEB-TACE**	0.45 (0.12, 1.52)	4.50 (0.19, 190.02)	1.89 (0.14, 24.60)	2.50 (0.18, 31.09)	2.36 (0.26, 19.75)	0.85 (0.07, 10.46)
2.24 (0.66, 8.08)	**TACE**	9.94 (0.58, 353.40)	4.27 (0.44, 42.24)	5.55 (0.56, 56.70)	5.28 (0.93, 30.63)	1.90 (0.24, 15.87)
0.22 (0.01, 5.30)	0.10 (0.00, 1.71)	**TACE+EBRT**	0.41 (0.01, 16.81)	0.54 (0.01, 21.65)	0.53 (0.01, 15.33)	0.19 (0.00, 7.17)
0.53 (0.04, 7.19)	0.23 (0.02, 2.26)	2.42 (0.06, 192.73)	**TACE+HIFU**	1.34 (0.05, 35.35)	1.27 (0.07, 22.96)	0.44 (0.02, 9.35)
0.40 (0.03, 5.46)	0.18 (0.02, 1.78)	1.84 (0.05, 110.87)	0.75 (0.03, 18.41)	**TACE+PEI**	0.94 (0.06, 16.57)	0.34 (0.02, 8.28)
0.42 (0.05, 3.89)	0.19 (0.03, 1.08)	1.89 (0.07, 89.44)	0.78 (0.04, 14.13)	1.06 (0.06, 17.73)	**TARE_90Y**	0.36 (0.02, 5.80)
1.17 (0.10, 13.69)	0.53 (0.06, 4.13)	5.26 (0.14, 342.35)	2.26 (0.11, 48.82)	2.95 (0.12, 65.87)	2.78 (0.17, 45.38)	**TEA**

* DEB-TACE: drug-eluting beads-transcatheter arterial chemoembolization; TACE: transcatheter arterial chemoembolization; EBRT: external-beam radiation therapy; HIFU: high intensity focused ultrasound; PEI: percutaneous ethanol injection; TARE-90Y: yttrium-90 radioembolization; TEA: transarterial ethanol ablation.

**Table 3:** PR of eight minimally invasive surgeries in unresectable HCC according to the network meta-analysis using odds ratios (ORs) and corresponding 95% credible intervals (CrIs)

**DEB-TACE**	0.61 (0.20, 1.74)	3.53 (0.32, 34.46)	0.93 (0.07, 10.23)	0.28 (0.03, 2.88)	1.12 (0.11, 8.73)	0.74 (0.15, 3.51)	0.63 (0.03, 10.20)
1.63 (0.58, 5.08)	**TACE**	5.66 (0.66, 45.59)	1.50 (0.16, 14.16)	0.46 (0.06, 3.77)	1.81 (0.26, 11.14)	1.21 (0.41, 4.05)	1.03 (0.07, 14.77)
0.28 (0.03, 3.10)	0.18 (0.02, 1.52)	**TACE+EBRT**	0.27 (0.01, 6.23)	0.08 (0.00, 1.60)	0.32 (0.02, 4.89)	0.21 (0.02, 2.63)	0.18 (0.01, 5.58)
1.07 (0.10, 13.39)	0.67 (0.07, 6.10)	3.76 (0.16, 82.86)	**TACE+HIFU**	0.30 (0.02, 6.36)	1.18 (0.06, 19.92)	0.80 (0.07, 10.45)	0.68 (0.02, 19.78)
3.59 (0.35, 38.27)	2.20 (0.27, 17.77)	12.51 (0.63, 233.52)	3.31 (0.16, 65.73)	**TACE+PEI**	4.07 (0.21, 58.33)	2.68 (0.26, 29.01)	2.25 (0.07, 64.38)
0.90 (0.11, 9.13)	0.55 (0.09, 3.91)	3.09 (0.20, 53.86)	0.85 (0.05, 16.49)	0.25 (0.02, 4.73)	**TACE+SOR**	0.68 (0.08, 6.83)	0.56 (0.02, 14.89)
1.35 (0.29, 6.49)	0.83 (0.25, 2.44)	4.69 (0.38, 48.45)	1.26 (0.10, 15.27)	0.37 (0.03, 3.83)	1.48 (0.15, 12.07)	**TARE-90Y**	0.85 (0.04, 14.95)
1.60 (0.10, 28.84)	0.97 (0.07, 14.08)	5.56 (0.18, 165.28)	1.47 (0.05, 50.56)	0.44 (0.02, 13.84)	1.77 (0.07, 45.74)	1.18 (0.07, 24.57)	**TEA**

* DEB-TACE: drug-eluting beads-transcatheter arterial chemoembolization; SOR: sorafenib; TACE: transcatheter arterial chemoembolization; EBRT: external-beam radiation therapy; HIFU: high intensity focused ultrasound; PEI: percutaneous ethanol injection; TARE-90Y: yttrium-90 radioembolization; TEA: transarterial ethanol ablation.

**Table 4:** SD of eight minimally invasive surgeries in unresectable HCC according to the network meta-analysis using odds ratios (ORs) and corresponding 95% credible intervals (CrIs)

**DEB-TACE**	1.10 (0.28, 4.09)	0.40 (0.02, 8.20)	0.40 (0.01, 9.64)	1.30 (0.05, 41.04)	2.94 (0.24, 41.56)	1.30 (0.18, 9.24)
0.91 (0.24, 3.62)	**TACE**	0.37 (0.02, 5.61)	0.36 (0.02, 6.50)	1.19 (0.06, 27.30)	2.67 (0.34, 25.22)	1.19 (0.29, 5.01)
2.47 (0.12, 58.70)	2.68 (0.18, 45.42)	**TACE+EBRT**	1.00 (0.02, 52.42)	3.25 (0.05, 207.23)	7.06 (0.25, 281.84)	3.21 (0.15, 77.92)
2.50 (0.10, 67.57)	2.75 (0.15, 51.88)	1.00 (0.02, 57.23)	**TACE+HIFU**	3.22 (0.05, 243.27)	7.20 (0.21, 308.07)	3.21 (0.13, 89.04)
0.77 (0.02, 20.63)	0.84 (0.04, 17.24)	0.31 (0.00, 19.14)	0.31 (0.00, 21.53)	**TACE+PEI**	2.31 (0.05, 102.92)	0.99 (0.03, 29.37)
0.34 (0.02, 4.17)	0.37 (0.04, 2.95)	0.14 (0.00, 4.04)	0.14 (0.00, 4.74)	0.43 (0.01, 19.15)	**TACE+SOR**	0.45 (0.03, 5.26)
0.77 (0.11, 5.53)	0.84 (0.20, 3.40)	0.31 (0.01, 6.60)	0.31 (0.01, 7.92)	1.01 (0.03, 29.25)	2.24 (0.19, 34.70)	**TARE-90Y**

* DEB-TACE: drug-eluting beads-transcatheter arterial chemoembolization; SOR: sorafenib; TACE: transcatheter arterial chemoembolization; EBRT: external-beam radiation therapy; HIFU: high intensity focused ultrasound; PEI: percutaneous ethanol injection; TARE-90Y: yttrium-90 radioembolization; TEA: transarterial ethanol ablation.

**Table 5:** PD of seven minimally invasive surgeries in unresectable HCC according to the network meta-analysis using odds ratios (ORs) and corresponding 95% credible intervals (CrIs)

**DEB-TACE**	3.04 (1.07, 10.57)	0.11 (0.01, 2.44)	1.28 (0.11, 18.66)	2.19 (0.26, 17.71)	1.63 (0.22, 12.32)	2.83 (0.56, 13.98)
**0.33 (0.09, 0.94)**	**TACE**	**0.04 (0.01, 0.52)**	0.42 (0.04, 4.23)	0.71 (0.11, 3.90)	0.54 (0.10, 2.52)	0.93 (0.26, 2.57)
9.05 (0.41, 405.50)	**27.10 (1.93, 1230.17)**	**TACE+EBRT**	11.94 (0.32, 923.49)	19.67 (0.65, 1176.68)	14.53 (0.59, 893.34)	**25.09 (1.22, 1280.41)**
0.78 (0.05, 8.97)	2.38 (0.24, 23.82)	0.08 (0.01, 3.09)	**TACE+HIFU**	1.68 (0.09, 26.23)	1.28 (0.07, 18.61)	2.20 (0.16, 25.12)
0.46 (0.06, 3.80)	1.41 (0.26, 9.52)	0.05 (0.01, 1.53)	0.59 (0.04, 11.37)	**TACE+PEI**	0.75 (0.07, 8.81)	1.30 (0.16, 10.91)
0.61 (0.08, 4.49)	1.85 (0.40, 10.42)	0.07 (0.01, 1.69)	0.78 (0.05, 14.23)	1.33 (0.11, 13.70)	**TACE+SOR**	1.75 (0.23, 12.39)
0.35 (0.07, 1.78)	1.08 (0.39, 3.88)	**0.04 (0.01, 0.82)**	0.45 (0.04, 6.28)	0.77 (0.09, 6.36)	0.57 (0.08, 4.31)	**TARE-90Y**

* DEB-TACE: drug-eluting beads-transcatheter arterial chemoembolization; SOR: sorafenib; TACE: transcatheter arterial chemoembolization; EBRT: external-beam radiation therapy; HIFU: high intensity focused ultrasound; PEI: percutaneous ethanol injection; TARE-90Y: yttrium-90 radioembolization.

**Table 6:** ORR of nine minimally invasive surgeries in unresectable HCC according to the network meta-analysis using odds ratios (ORs) and corresponding 95% credible intervals (CrIs)

**TACE**	1.79 (0.20, 12.50)	3.44 (0.29, 41.37)	4.03 (0.38, 63.63)	2.74 (0.83, 9.19)	1.42 (0.43, 5.33)	7.20 (0.65, 72.68)	1.92 (0.16, 22.10)
0.56 (0.08, 5.09)	**TACE+SOR**	1.93 (0.08, 57.91)	2.33 (0.10, 81.79)	1.55 (0.16, 19.74)	0.81 (0.08, 10.40)	4.02 (0.18, 111.07)	1.08 (0.05, 27.91)
0.29 (0.02, 3.49)	0.52 (0.02, 11.99)	**TACE+HIFU**	1.20 (0.04, 45.77)	0.79 (0.05, 13.19)	0.42 (0.03, 7.44)	2.08 (0.06, 62.72)	0.57 (0.02, 18.80)
0.25 (0.02, 2.65)	0.43 (0.01, 10.17)	0.83 (0.02, 26.83)	**TACE+PEI**	0.68 (0.04, 10.21)	0.35 (0.02, 5.61)	1.73 (0.05, 52.42)	0.46 (0.01, 14.33)
0.37 (0.11, 1.20)	0.65 (0.05, 6.32)	1.26 (0.08, 20.33)	1.47 (0.10, 28.10)	**DEB-TACE**	0.52 (0.10, 3.09)	2.63 (0.17, 36.12)	0.70 (0.04, 10.54)
0.70 (0.19, 2.33)	1.23 (0.10, 12.32)	2.41 (0.13, 39.14)	2.88 (0.18, 52.65)	1.91 (0.32, 10.32)	**TARE-90Y**	4.99 (0.30, 68.79)	1.35 (0.08, 19.55)
0.14 (0.01, 1.55)	0.25 (0.01, 5.45)	0.48 (0.02, 15.58)	0.58 (0.02, 19.57)	0.38 (0.03, 5.86)	0.20 (0.01, 3.37)	**TACE+EBRT**	0.27 (0.01, 7.79)
0.52 (0.05, 6.37)	0.92 (0.04, 21.01)	1.76 (0.05, 62.91)	2.19 (0.07, 76.26)	1.42 (0.09, 23.59)	0.74 (0.05, 13.32)	3.71 (0.13, 129.28)	**TEA**

* DEB-TACE: drug-eluting beads-transcatheter arterial chemoembolization; SOR: sorafenib; TACE: transcatheter arterial chemoembolization; EBRT: external-beam radiation therapy; HIFU: high intensity focused ultrasound; PEI: percutaneous ethanol injection; TARE-90Y: yttrium-90 radioembolization; TEA: transarterial ethanol ablation.

Another interesting trend is that DEB-TACE and TACE+EBRTshow a higher PD ratethan TACE. Therefore, we suspected that TACE+EBRT may provide HCC patients with favorable results with respect to disease progression control whereas single TACE may not provide such effectiveness.

### Cumulative ranking

Table 7 shows the relative ranking of eight minimally invasive treatments which are widely applied in clinical practices for unresectable HCC. As suggested by the corresponding SUCRA values, TACE+EBRT appeared to have the highest ranking in CR (76.50%), PR (88.50%), PD (95.33%) and ORR (81.13%). TACE+SOR exhibited the highest ranking probability (79.14%) with respect to SD. All of this evidence enabled us to conclude that TACE+EBRTmay be the most appropriate treatment for managing patients with unresectable HCC.

**Table 7:** Relative ranking of nine minimally invasive surgeries in unresectable HCC assessed by using SUCRA values

Treatment	CR	PR	SD	PD	ORR
DEB-TACE	38.67%	**60.88%**	46.57%	**67.33%**	61.25%
TACE	8.17%	37.50%	50.00%	19.50%	21.88%
TACE+EBRT	**76.50%**	**88.50%**	26.14%	**95.33%**	**81.13%**
TACE+HIFU	58.33%	55.13%	25.14%	**54.67%**	**63.75%**
TACE+PEI	**66.00%**	20.63%	55.14%	38.00%	**68.50%**
TACE+SOR	-	**62.50%**	**79.14%**	48.00%	46.25%
TARE-90Y	**66.83%**	48.25%	**56.86%**	26.33%	38.50%
TEA	33.83%	43.63%	-	-	47.75%

* CR: complete response; PR: partial response; SD: stable disease; PD: progression disease; ORR: objective response rate; DEB-TACE: drug-eluting beads-transcatheter arterial chemoembolization; SOR: sorafenib; TACE: transcatheter arterial chemoembolization; EBRT: external-beam radiation therapy; HIFU: high intensity focused ultrasound; PEI: percutaneous ethanol injection; TARE-90Y: yttrium-90 radioembolization; TEA: transarterial ethanol ablation.

## DISCUSSION

As one of the most common malignant tumors, the mortality ofHCC is extremely high and we have witnessed a large number of families struggling with this disease [26, 27]. In this study, we collected data from 17 studies with respect to eight minimally invasive surgical approaches in order to assess their short-term efficacy and safety. Our research objective is to rank the corresponding surgical approaches by using theelegant approach of network meta-analysis. It appears that TACE+EBRT was more effective than the other seven surgical approaches. Moreover, TACE+SOR, DEB-TACE,TACE+HIFU, TACE+PEI and TARE-90Y exhibited stronger performance than expected.

TACE has been recommended for patients who are not suitable for surgeries [28]. However, arterial occlusion in tumor tissues may trigger hypoxia and hence contribute to the over-expression of hypoxia-inducible factor 1 (HIF-1α) and vascular endothelial growth factor (VEGF) [29, 30].While the over-expression of HIF-1α could cause tumor progression, invasion and metastasis [31, 32], VEGF inhibits the apoptosis of vessel endothelial cells and promotes proliferation [33, 34] and it triggers tumor progression by stimulating the formation of blood vessels [35]. Although our study demonstrated the short-term effects of these surgical approaches, whether these effects remain strong in the long term should be further verified [36, 37]. As suggested by previous studies, introducing EBRT into TACE was able to enhance the effectiveness of TACE alone and hence improve the overall survival status of HCC patients [23, 38, 39]. Besides that, both direct and indirect evidence from the network meta-analysis reached a conclusion that TACE+EBRT were more effective than TACE alone with respect to CR, PR and ORR. By contrast, TACE had better performance than TACE+EBRT with respect to SD and PD.On top of that, TACE+EBRT exhibited compelling results in PR, PD, CR, ORR and DCR in comparison to the other seven minimally invasive surgeries.

One major issue of TACE was contributed by the over-expression of VEGF and HIF-1α which result in tumor progression, invasion and metastasis [29, 30, 40].SOR is a multikinase inhibitor which is able to suppress tumor cell proliferation by targeting theVEGF receptor and/or the PDGF receptor [41, 42]. Our study supported the notion that introducing SOR into TACE could significantly enhance the effectiveness of TACE which was reflected by the improved PR and SD. This trend may be explained by the fact that SOR is likely to suppress the expression of VEGF and hence reduce the side effects resultedfrom TACE. Additionally, DEB-TACE also exhibited promisingresults in the SUCRA ranking. DEB-TACE is able to alleviate the local embolization resulted from HCC lesions and prolong the release of chemotherapeutic agents into adjacent tissues [43, 44].Furthermore, DEB-TACE is able to alleviate the symptoms of HCC by releasing some antitumor lesion factors so that the side effects of TACE can be prevented [45–47].As suggested by the corresponding ranking of SUCRA, TARE-90Y, TACE+HIFU and TACE+PEI all displayed desirable results compared to TACE alone in most of the aspects. It is known that 90Y is a pure β emitter which stimulates the physical half-life of zirconium-90 to approximately 64.1 hours and such a stimulation enables it to have some excellent properties being an effective transarterial liver-directed agent [48]. More importantly, TARE-90Y is able to release radioactive particles into the liver artery without significant arterial occlusion [49] and HIFU perhaps is able to induce the complete coagulative necrosis of tumor without affecting adjacent tissues and structures [50].On the other hand, TACE+PEI have been considered as an appropriate option for small HCC lesions provided that the diameter of tumorshould be less than 3 cm and such a criterion has reduced the popularity of TACE+PEI in clinical practices [51].

Nevertheless, some limitations may have uncertain impact on theoverall conclusions. For instance, four surgeries (TACE+HIFU, TACE+PEI, TACE+EBRT, TEA) contained only one eligible trial and thus the assessment of these approaches may not be as accurate as those approaches in which a large number of trials were included. Inherent difference in studies and trials such as study design, assessment approach and surgical procedures may also have influenceon the overall results. Finally, we intentionally removed some studiesin which side effects were not mentioned and such missing studies may contain key information with respect to the efficacy and safety of these surgeries.

For summary, TACE+EBRT exhibited the most compelling results in comparison to the other seven minimally invasive surgeries. Futures studies should investigate the long-term effects of these minimally invasive surgeries on HCC patients by designingappropriate following-up studies. This study may provide sensible perspectives with respect to choosing the appropriate surgical approaches for individual HCC patients.

## MATERIALS AND METHODS

### Search strategy

Key terms such as”unresectable hepatocellular carcinoma”, “transcatheter arterial chemoembolization”, “sorafenib”, “high intensity focused ultrasound”, “percutaneous ethanol injection”, “drug-eluting beads”, “yttrium-90 radioembolization”, “external-beam radiation therapy”, “transarterial ethanol ablation” and “randomized controlled trial” (dated up to May of 2016) were used to retrieve academic articles from PubMed, Embase and Cochrane Library. No muti-arm trials were found. Additionally, a manual search of primary and secondary publication references was conducted to identify additional relevant studies.

### Inclusion and exclusion criteria

In the meta-analysis, studies that met the following criteria were included: (i) randomized controlled trials; (ii) one or more of the following interventions were involved in the trials: TACE, TACE+SOR, SOR, TACE+HIFU, TACE+PEI, DEB-TACE, TARE-90Y, TACE+EBRT and TEA; (iii) patients involved were over 18yearsold with unresectableHCC; (iv) no operative interventions had been conducted before the trials were conducted; (v) one or more of the following outcomes were assessed: complete response (CR), partial response (PR), stable disease (SD), progression disease (PD) and objective response rate (ORR).

However, studies were excluded if any of the following conditions were satisfied: (i) patients involved failed to perform normal activities or did not have normal functioning kidneys, lungs or heart; (ii) treatments for unresactableHCC, such as surgery, medication, radiotherapy and others, had been given; (iii) unqualified literatures which did not have academic integrity (e.g. incorrectly matched case-control studies).

### Data extraction and outcome measures

The corresponding data was extracted by two reviewers independently, and then were entered into Excel spreadsheets that were created by the third reviewer. The data were extracted including the author, region, year, treatment, size of sample and endpoints. Disagreements between the two reviewers, if any, were resolved by consensus or with the help of the third reviewer.

Main outcome measures include: (i) CR: all of clinically visible pathological features were eliminated for more than 4weeks and tumor marker was monitored without new pathological changes; (ii) PR: sum of the maximum diameter of tumor clinical lesions had decreased by more than 30% or the product of the maximum diameter and maximum vertical diameter had decreased by more than 50%, and had lasted for more than 4 weeks; (iii) PD: sum of the maximum diameter of tumor clinical lesions had increased by more than 20%, the product of two maximum diameters had increased by 25%, or new lesions had developed; (iv) SD: reduced sum or product of the two maximum diameters were below the standard of PR, or the increased amount was below the standard of PD; (v) ORR = CR+PR: the sum of complete response and partial response cases.

### Statistical analysis

Initially, we performed direct meta-analysis with R software (V3.2.1). Binary outcomes were then compared in terms of odds ratios (OR) and 95% confidence interval (CI). Inter-study heterogeneity was identified using the Q test and *I*^2^ test. If there was no significant heterogeneity among studies, a fixed-effects model was selected for analysis; otherwise a random-effects model was selected for analysis [52]. Next, network meta-analysis was performed by using the GEMTC (V0.6) package and the corresponding results were combined with those obtained from the DerSimonian-Laird random-effects model and Monte Carlo Markov Chain (MCMC). We used R software (V3.2.1) in order to produce the surface under the cumulative ranking curve (SUCRA) and calculated the ranking of different interventions [53]. For each intervention, efficacy and safety outcomes were ranked by the SUCRA: a higher value of SUCRA indicated a higher ranking. All the calculations were conducted by using R software (V3.2.1), GEMTC package (V0.6) and Open BUGS (V3.4.0).
